# Ethics of non-oncological fertility preservation from the perspective of the four principles of bioethics

**DOI:** 10.3389/frph.2026.1837791

**Published:** 2026-05-20

**Authors:** Silviya Aleksandrova-Yankulovska, Marcin Orzechowski, Katharina Hancke, Karin Bundschu, Florian Steger

**Affiliations:** 1 Institute of the History, Philosophy and Ethics of Medicine, Ulm University, Ulm, Germany; 2 Department of Gynaecology and Obstetrics, Ulm University, Ulm, Germany

**Keywords:** endometriosis, non-oncological fertility preservation, principles of bioethics, social egg freezing, transgender individuals

## Abstract

**Introduction and aims:**

Fertility preservation refers to the cryopreservation of gametes, embryos, or gonadal tissue before age, disease, or medical treatment affects reproductive capacity. Fertility preservation techniques were originally developed for cancer patients. Over time, more population groups have been interested in using these technologies—such as endometriosis patients, transgender individuals, and healthy women opting for social egg freezing. While ethics in oncological fertility preservation is well studied, the ethics of non-oncological cases remains less explored. The aim of our research is to identify the ethically distinctive features of non-oncological fertility preservation in women with endometriosis, women opting for social egg freezing, and transgender individuals.

**Materials and methods:**

We performed an ethical analysis of scientific literature on ethical aspects of non-oncological fertility preservation, using the four principles of bioethics. The literature review encompassed four databases: Science Direct, Web of Science, PubMed, and BELIT. After the exclusion of ineligible records, 56 scientific articles were analysed further.

**Results:**

In relation to reproductive autonomy and informed consent, we found challenges such as decisional pressure, provision of proper information, and questionable ability to make future-oriented decisions. The principle of beneficence encompasses various notions of medical, psychological, social, and personal benefit, most notably the benefits of safeguarding genetic parenthood and prevention of future regret. Concerning the principle of non-maleficence, non-oncological fertility preservation was accompanied by medical, psychological, and social risks, such as delay in therapy, misinformation, and false hope. The principle of social justice highlighted problems like unequal access, lack of health insurance coverage, and gender discrimination concerns.

**Conclusion:**

Our study has provided identification and ethical analysis of the issues linked to fertility preservation in women with endometriosis, women opting for social egg freezing, and transgender individuals. The underlined differences in ethical sense between non-oncological and oncological fertility preservation support the need for a tailored ethical approach to these patients. Future research is needed to examine how ethical challenges evolve as technologies develop and clinical use expands.

## 1 Introduction

Fertility preservation refers to medical interventions used to cryopreserve gametes, embryos, or gonadal tissue before they are affected by time, disease, or medical treatment (1). These techniques were originally developed for cancer patients facing fertility loss from gonadotoxic anti-cancer therapies (2). Over time, more groups began using fertility preservation. These include women with endometriosis (3–5), transgender individuals (6), and women choosing social egg freezing (7). We further refer to these cases as “non-oncological fertility preservation”.

The practice with the fertility preservation techniques and the chances of a successful outcome are variable. Sperm, oocytes, and embryos freezing are already well-established methods (8). However, freezing ovarian and testicular tissue is relatively new, and many clinics lack reliable experience (8). Additionally, the rate of utilisation of frozen gametes appears to be low. In a study of Jones et al. (9) cryopreserved sperm was only utilised by one in nine (11%) transwomen. In a study of Barrett et al. (10) none of 44 young adult transmen returned to use their cryopreserved oocytes. Between 10% and 16% of women use their frozen eggs in cases of social egg freezing (11–13), while millions of cryopreserved oocytes remain in storage (14), which raises questions about cost-effectiveness. The ethical issues accompanying fertility preservation techniques have been studied mainly in cancer patients. The ethics of oncological fertility preservation covers a broad spectrum of considerations such as reproductive autonomy (15); decisional capacity of patients-children (16); the challenges of informed consent, particularly in case of posthumous reproduction (17); benefits of reassuring survivorship (18) and imagining future as a parent (19); risks of delay in cancer treatment (20) and creating false hope (21); as well as social justice concerns about unequal funding schemes (22), to name a few. Regarding the fate of reproductive biomaterial, empirical findings show that female cancer patients, for example, generally accept the use of reproductive cells for reproductive purposes, but they tend to reject their use in research unrelated to fertility preservation (23). Against this background, the ethics of non-oncological fertility preservation remain underinvestigated.

Our research aims to identify the ethically distinctive features of non-oncological fertility preservation.

To achieve this aim, we perform an ethical analysis of the scientific literature on the ethical aspects of fertility preservation in women with endometriosis, women wishing social egg freezing, and transgender individuals through the four principles of bioethics (24).

## 2 Materials and methods

We conducted an ethical analysis based on a literature search. Such an approach is frequently used to analyse ethical issues connected to new technological developments in medical practice (25).

The search was conducted in four databases: Science Direct, Web of Science, PubMed, and BELIT in the period April 2024–March 2026. The search algorithm combined the following keywords: “fertility preservation” OR “fertility protection” AND “ethics” OR “ethical issues”. The terms “fertility protection” and “fertility preservation” were used in the analysed papers with the same meaning. Therefore, we use only the term “fertility preservation” in this paper.

We applied the following inclusion criteria: mention of non-oncological fertility preservation, clear ethical focus, publication date between 2000 and 2025, English language, and full-text availability. We excluded studies that had no specific mention of fertility preservation, no ethical focus, no link to humans, only legal or clinical discussion, or focus only on oncological cases, missing full text.

**Figure 1:**
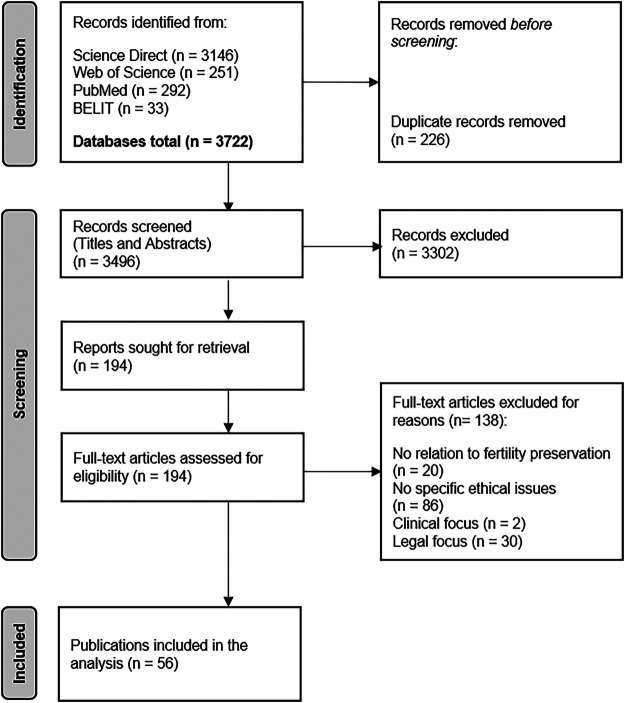
Flow diagram of the literature search process.

The search algorithm found 3.722 records. After removing 226 duplicates, the titles and abstracts were screened. Following the screening procedure, 3.302 records were excluded for not meeting the inclusion criteria. Ultimately, 194 publications were classified as relevant, retrieved, and read in full. Of these, 138 articles were additionally excluded based on the same criteria. The final number of publications included in our analysis was 56 (Figure 1). The publications were distributed by user groups as follows: publications concerning ethical issues of fertility preservation in women with endometriosis—5; in women opting for social egg freezing—32; in transgender individuals—19.

The selected publications were subjected to thematic analysis (26). Thematic analysis is a qualitative analytic method for identifying, analysing, and reporting common patterns (themes) in narratives and text materials (26). The thematic analysis was performed by the first author in accordance with Braun's and Clarke's latest recommendations for single coder (27). The results were discussed in the authors' team. Our interdisciplinary team comprise of ethicist and public health expert (S.A.Y.), ethicist and political scientist (M.O.), physicians-gynaecologists (K.H. and K.B.), and physician and expert in ethics and history of medicine (F.S.). A combination of inductive and deductive thematic analysis was applied. First, the inductive thematic analysis of the full text of the selected articles was conducted. The coding process was performed manually and involved highlighting relevant ethical issues in the articles. Then, the identified codes were assigned to predefined themes of the four bioethical principles, i.e., autonomy, beneficence, non-maleficence, and social justice (24).

Finally, an ethical analysis through the prism of the four bioethical principles by Beauchamp and Childress (24) was carried out. This approach has been chosen as most popular in medical ethics and well accepted among practicing healthcare professionals, which corresponds to the expected main audience of our research. It allows consideration of different perspectives, in which individual aspects (autonomy, beneficence, non-maleficence) are combined with more general aspects (social justice) of patient care.

## 3 Results

In this section, we present the results of the thematic analysis. Thematic analysis found common ethical issues across all three groups. It also showed specific ethical issues for each group. These are shown in Table 1. We linked the results to the four bioethical principles to support the ethical analysis.

**Table 1:** Common and specific ethical issues identified in the literature by patient groups (in columns) and bioethical principles (in rows).

Bioethical principle		Women with endometriosis	Social egg freezing	Transgender individuals
Autonomy	*Common issues for the three groups*	*Enhanced autonomy* (27) *Decisional pressure* (13, 29) *Additional information tools* (30, 31) *2-stage informed consent* (32)		
	Specific issues by group	Not specifically addressed in the analysed articles	Reproductive independence (15) Quality of informed consent (32)	Ability to make future-oriented decisions (16) Insufficient provider knowledge (30)
Beneficence	*Common issues for the three groups*	*Reproductive insurance* (33) *Genetic parenting* (28, 34) *Prevention of regret* (35)		
	Specific issues by group	Medical benefit (3)	Lower risks of chromosomal abnormalities (28) Preparedness for parenthood (28) “Exploitative benefit” (13)	Better quality of life (33)
Non-maleficence	*Common issues for the three groups*	*Misinformation* (13, 31) *False hope* (3, 36)		
	Specific issues by group	Emotional burden (35) Financial burden (35)	Health risks for the women (28, 38) Social and emotional difficulties for the child (21, 28) Disturbed intergenerational contract (39) Manipulation of decision time (40)	Delay in treatment (35) Gender dysphoria exacerbation (41, 42) Discrimination of offspring (6) Challenged notion of family (43, 44) Decisional regret (35)
Social justice	*Common issues for the three groups*	*Equal access* (45) *Coverage* (46)		
	Specific issues by group	Unclear cost-effectiveness (37) Impact on public healthcare systems (47)	“Gender equaliser” misrepresentation (48)	Objection of health professionals (49) Unequal research attention (50) Adoption and surrogacy challenges (16, 41)

### 3.1 Autonomy

Autonomy was discussed in terms of the concept of reproductive autonomy and the various elements of informed consent.

Reproductive autonomy is the capacity to act in accordance with one's own reasons and values when it comes to one's reproductive choices (15). Fertility preservation techniques enhance reproductive autonomy by offering more parenthood options (28). But it may also limit autonomy by creating pressure to use these options (15).

The principle of respect for autonomy finds expression in informed consent and its elements: capacity for self-determination (capacity for autonomous choice), freedom of decision-making (voluntariness), and information provision (disclosure) (24).

Within the studied groups, the capacity for self-determination was questioned for transgender youth. Their intellectual, emotional, and social immaturity (50, 51) was seen as limiting their *ability to make future-oriented decisions* (16).

Freedom of decision-making can be affected by *decisional pressure*, i.e., outside pressure on patients to choose a certain option. It can be experienced by healthy women whose employers covered the financial costs for egg freezing (13, 15, 29, 52–54) or by transgender youth on the side of their parents (55).

Adequate information provision is essential for informed consent, requiring providers to possess proper knowledge. In transgender care, *healthcare providers* were found to have insufficient *knowledge* (30). All studied groups would benefit from the availability of *additional information tools* (30, 31, 56–58).

Fertility preservation requires a unique *2-stage informed consent process* (32). The first stage is the decision to harvest gametes, while the second is the decision to use them for reproduction. This increases the need for comprehensive information (59), especially regarding the likelihood of achieving a child delivery (21).

### 3.2 Beneficence

The literature discusses different types of benefit from fertility preservation: medical benefit, psychological benefit, social benefit, and personal benefit.

Medical benefit: Fertility preservation helps women with endometriosis, especially those with bilateral endometriomas or early diagnosis, due to a higher risk of recurrence (3). For women choosing social egg freezing, the use of younger oocytes is associated with reduced rates of miscarriage and chromosomal abnormalities after age 35 (28).

Psychological benefit: For all groups, fertility preservation acts as “*reproductive insurance*”, giving a sense of security and relief (33). Healthy women may also avoid “panic partnering” (60) and wait until they are *better prepared for parenthood* (28).

*Social benefit:* In social egg freezing, widely discussed is the “*exploitative benefit*”. This means women can remain longer in the workforce without concern about age-related fertility decline (“biological clock”) (13).

*Personal benefit:* Fertility preservation offers the chance for *genetic parenthood* when fertility is endangered (28, 34). It may, therefore, *prevent patients' regret* in the future when they would like to have children (35). This also *improves quality of life*, especially noted in transgender-focused publications (33).

### 3.3 Non-maleficence

The literature discusses medical, psychological, and social risks of fertility preservation.

*Medical risks:* For women, fertility preservation involves risks like ovarian hyperstimulation syndrome (28), surgical complications (38), and late pregnancy risks (61). In trans women, it may *delay the initiation of gender dysphoria treatment* (35). Collecting reproductive biomaterial in all transgender individuals might lead to *dysphoria exacerbation* (41, 42, 49) and increased suicidal risk (62).

*Psychological risks:* The main risk is *misinformation*, caused by lack of data on clinical outcomes of fertility preservation, lack of unified procedure standards for all patient groups (13), misleading advertising (31), cost-benefit misrepresentation for profit (29), or inadequate preparation of providers to advise on fertility preservation (30, 63).

In endometriosis patients, preserving reproductive biomaterial can be *emotionally burdening* and cause feelings of fear and re-connection with their disease (37). Children born after social egg freezing risk *social and emotional difficulties* associated with advanced parental age (21, 28).

The main harm linked to non-maleficence is *false hope*. This comes from low success rates of fertility preservation and weak evidence that fertility preservation will eventually result in child delivery (3, 21, 34). Misinformed patients not only risk an inability to have the desired family (31) but plan their future on false beliefs (29) and a sense of security (28). They may omit realistic options such as adoption or earlier pregnancy (3, 37).

*Social risks:* Delayed motherhood through social egg freezing *disturbs the intergenerational contract*. Older mothers and their children may not support each other (38). Grandparents may also miss child-raising (39). Social egg freezing *manipulates decision time* (40), moving it into the future when women eventually decide about the use of the frozen eggs. Thus, egg freezing alone doesn't solve impending issues of pregnancy, partnership, and career (56).

For transgender individuals, risks range from *discrimination of future offspring* (6) to a *challenged notion of family* (43, 44). Transgender adolescents, who reject fertility preservation in pursuit of transitional hormone therapy, risk experiencing *decisional regret* later in life (35).

Concerning endometriosis patients, the *financial burden* of fertility preservation and consequent artificial reproduction is high. This is especially true for young women early in their careers (37).

### 3.4 Social justice

Our study found several linked views of social justice: equal access, coverage concerns, gender discrimination, and cost-effectiveness.

*Equal access and coverage:* One of the biggest issues is access to fertility preservation. Some authors urge healthcare professionals to advocate for universal insurance coverage (46) because fertility preservation cost was identified as the main barrier (45). Some public health systems cover certain procedures, but funding rules vary widely. Patients risk unequal treatment also because of the different practices of the centers and the lack of uniform standards (64).

Transgender individuals face additional barriers due to their transgender identity conflicting with some physicians’ personal beliefs (6). To avoid the *conscientious objection on the side of healthcare professionals*, transgender individuals often hide their gender identity to receive care and relevant information (49, 65). They are also given lower priority in insurance policy and law (46, 66). The need for *further research on fertility preservation for transgender individuals* was additionally recognised (50). *Adoption or even surrogacy* are often proposed as better parenting options over the experimental and cost-ineffective fertility preservation techniques (16, 41). Transgender patients, however, often lack information about the challenges of these options, such as high costs, prolonged waiting times, and possible bias from surrogates, biological parents, or adoption agencies (16, 41).

*Gender discrimination:* Social egg freezing is often represented as a “*gender equalizer*” because it helps women manage “biological inequality” (48) and delay parenthood (13). But accepting oocyte freezing as a standard procedure may support gender bias because it normalises the different expectations towards young men and young women at work (67).

*Cost-effectiveness:* It is a main consideration in decisions about health insurance coverage. In endometriosis, limited data on cost-effectiveness hinders broad coverage (37). There is concern that making fertility preservation universally available to all women with endometriosis would strain health systems (47). A better option may be to offer fertility preservation to women with the highest likelihood of eventually using their frozen eggs in the future (68).

Cost-effectiveness is also low for social egg freezing (11–13). Thus, covering these costs would present a suboptimal allocation of scarce funds (69, 70). But without coverage, social egg freezing stays affordable only for a limited number of women (36, 71, 72).

## 4 Discussion

In this section, we discuss the results through the prism of the four bioethical principles.

### 4.1 Autonomy

Fertility preservation is believed to support reproductive autonomy in both oncological and non-oncological cases. However, more choices do not always mean more freedom. Cognitive psychology shows that too many choices can make decisions harder (31).

Patients may also face decisional pressure, which questions independence. For healthy women, this pressure can come from workplace policies that cover egg freezing, along with expectations to use it (29). But most of all, decisional pressure comes from biological time, societal, or future partner's expectations. These factors raise doubts about whether women today have more freedom than before in balancing work and pregnancy (29). The positive effect of egg freezing on women's autonomy depends on personal context, and not just access to the method (73). Thus, social egg freezing seems to have had rather a boomerang effect on autonomy, limiting it instead of enhancing it.

The availability of additional information tools contributes to better and independent information provision (30, 31, 56, 57). It can be in the form of professional and reliable online support, allowing patients to access information at any time, from any place, alone or shared with a partner or family member. The additional information tools can also contribute towards filling in the gap of insufficient provider knowledge (16, 30) and insufficient counseling (74).

Regarding the capacity for decision-making, we come to another concept less applicable to non-oncological fertility preservation. This is the right to open future. In pediatric oncology, this means parents make fertility choices for children, giving them a chance to decide later whether to use the preserved material (75). Among our study groups, this applies only to transgender youth, whose treatment in rare cases is initiated before reaching maturity. Although most transgender youth are sufficiently mature to participate in decision-making and provide assent, their ability to make future-oriented decisions would still be fragile. Therefore, fertility preservation gives them a chance to keep genetic parenthood as a future option (44).

In non-oncological cases, the informed consent process is complicated by limited experience with these groups and a lack of data on patient wishes and decision stability. Since this entails gaps in the available information, the question arises whether experiences of one user group can be transferred to another and used in the informed consent process. At least for transgender individuals, the World Professional Association for Transgender Health suggests using fertility data from cancer survivors in the consent process (41). What is of primary importance is the idea that informed consent is not merely a formal requirement but an integral part of the care process (76).

### 4.2 Beneficence and non-maleficence

When it comes to the application of new technologies in medicine, the balance between the principles of beneficence and non-maleficence always presents a challenge, because each technology involves both promises and risks. Fertility preservation affects patients' medical, psychological, and social well-being, with benefits and harms across all levels.

From a *medical perspective*, fertility preservation techniques, especially in women, are associated with many risks against uncertain benefits. However, this quantification is of limited relevance when the desire to have genetically related children is a primary concern for the patients. The layers of harm and benefit are closely linked. One may reduce the impact of another. For example, to avoid future regret, a patient may accept lightheartedly all medical risks associated with egg retrieval and late pregnancy (61). Although the risks of late pregnancy are generally well known, they should not be underestimated in the informed consent process.

From a *social perspective*, employers should focus more on developing family-friendly policies to keep women employees instead of funding egg freezing (29). After all, social egg freezing touches upon a deeply personal area of decision-making, and employer policies of financial coverage of egg freezing are seen as oppressive (29).

At the level of *psychological effects* of fertility preservation technologies, the most striking contrast is between the feeling of relief (33) and the risk of false hope (21) because the latter makes the former meaningless. If a patient feels secure because of fertility preservation, but fails to have a baby, then the relief turns out false. The trust built with doctors may collapse, replaced by blame for the failure. Therefore, special precautions should be made to avoid the risk of false hope.

Informed consent is the best ethical tool to balance these principles, and full and adequate information is essential. Thus, the informed consent process regarding fertility preservation in clinical practice should be strengthened and case-specific. Equal consideration should be given to all parenting options, including adoption and aiming at an earlier pregnancy, especially for women with endometriosis and healthy women opting for social egg freezing (3). Patients must be informed about legal and other barriers to each option. This helps to avoid misinformation and regret (13).

Fertility preservation discussions should always reflect each patient's personal situation. This ensures that benefits are maximized while harms are minimized.

### 4.3 Social justice

We identified many interpretations of social justice in fertility preservation.

*Equal access:* This can be improved by including fertility preservation in health insurance. Among the studied groups, the issue of equal access is most sensitive for transgender individuals. In fact, in 19 European countries, gender reassignment is either not allowed or transgender individuals cannot cryopreserve reproductive cells and tissues (77). This debate is complex, though. Supporting one group's access may open the door to others. For example, if coverage is granted to transgender individuals, it may also be requested for healthy women seeking social egg freezing (46). On a higher scale, there are also differences between developed and developing countries where even simple interventions are not always easily implemented, making social injustices to emerge in a variety of contexts (78).

*Gender discrimination:* This issue is strong in the social egg freezing debate. This debate, *per se*, tacitly confirms that unequal expectations for women and men in the labor market still exist (53). It is questionable, though, that denying funding is the best answer. When women must pay alone to protect their job stability, it adds to the problem of gender inequality. On the other side, there is an example of successful inclusion of egg freezing in health insurance. This is done in France, while funding from employers is forbidden (79). This example can be used in other countries where the demarcation of medical and non-medical reasons for fertility preservation is questioned.

*Insurance coverage gaps:* All studied groups fall behind public and health insurance coverage schemes for fertility preservation services. In endometriosis, the debate on coverage often focuses on the fears of high costs and the large number of patients (47). But this reasoning is flawed. Public health should help large groups in need. Fertility preservation for endometriosis patients is also reasonable due to current low fertility trends and the young age of affected women.

Instead of denying coverage, public health policies should focus on ways to improve the cost-effectiveness of fertility preservation techniques. Without going deeper into the multilayered arguments of this debate, which were well explored by other authors (15, 52, 67), we highlight that, given Europe's falling fertility rates, funding non-oncological fertility preservation deserves serious attention.

### 4.4 Limitations

Our study is planned as an ethical analysis, not a systematic literature review. Therefore, the algorithm of the literature search might not have identified all relevant publications on the topic and certain source selection bias is possible. However, the articles that were selected and analysed provide a good overview of the topic and support our ethical analysis. Additionally, our research was planned to cover publications on ethical issues of non-oncological fertility preservation. Thus, we do not incorporate real patient experiences and perspectives, which go beyond the aim of this research. Another limitation is the heterogeneity of the analysed groups, which restricted the intergroup comparisons. The majority of the sources consider a European perspective on ethical issues of fertility preservation, which may limit the generalizability of the findings to other healthcare systems.

## 5 Conclusion

Our study identified and analysed ethical issues in non-oncological fertility preservation for women with endometriosis, women choosing social egg freezing, and transgender individuals. This extends the discussion beyond oncofertility and helps to build a comprehensive picture of the ethics of fertility preservation as a whole.

**Figure 2:**
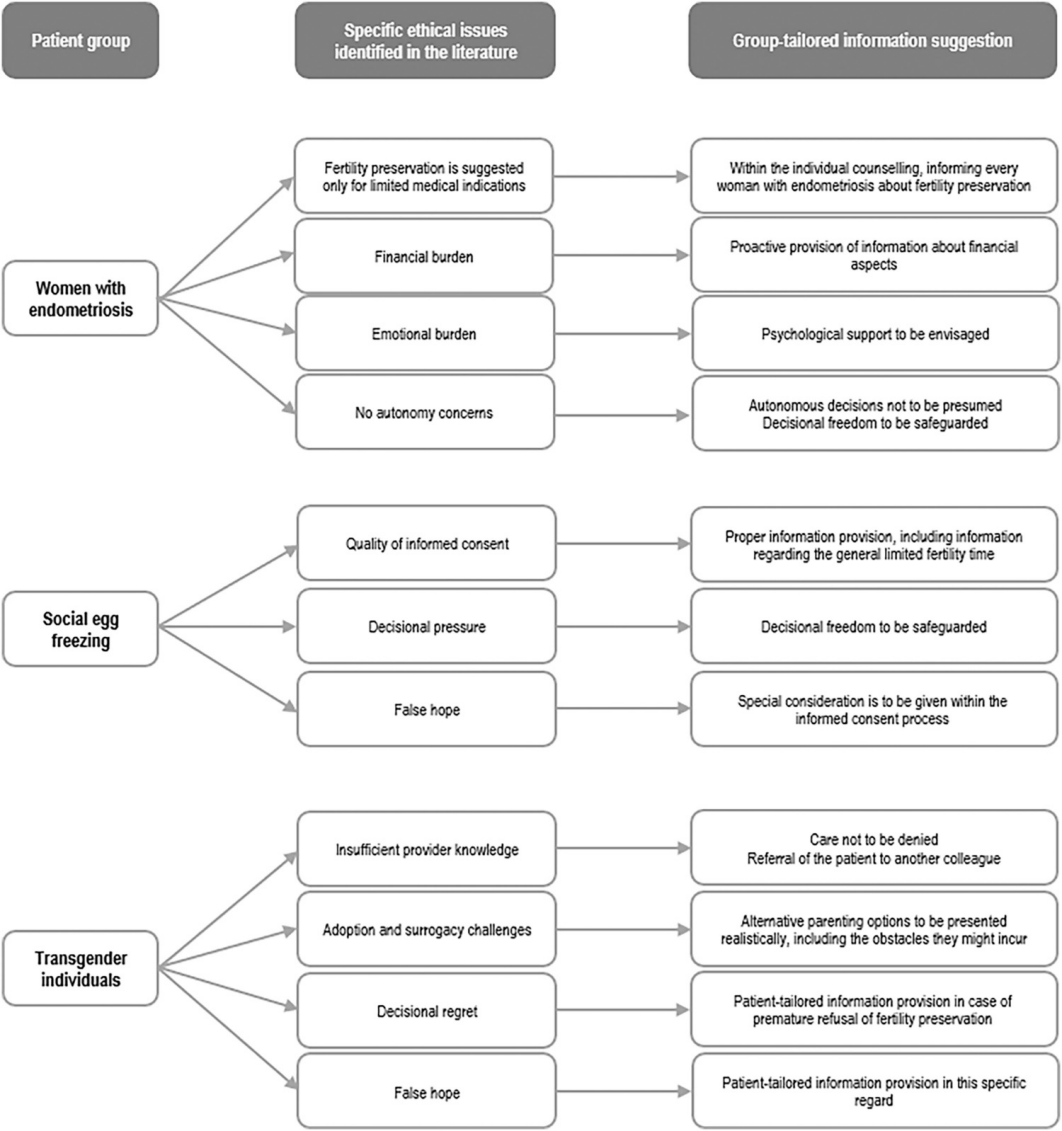
Overview of the group-tailored information suggestions.

We have underlined specific differences in ethical sense between non-oncological and oncological fertility preservation. Patients from both groups are treated by the same healthcare providers. But because their care raises different ethical concerns, they need different approaches and group-tailored information provision. On the basis of our results, such group-tailored information (Figure 2) should include:

1. *For women with endometriosis*: Within the framework of individual counselling to consider informing extensively every woman with endometriosis, not only the women with medically severe cases, about the possible benefits of fertility preservation and proactively to include information about its financial aspects. In cases of expected emotional burden, psychological support should be offered. Although we found no publications on autonomy in this group, patient's autonomous decision should not be presumed but safeguarded against possible decisional pressure on the side of family members.
2. *For women choosing social egg freezing*: The focus should be on proper information provision, including information regarding the general limited fertility time, and its understanding while safeguarding the independence of woman's decision. The personal situation of each woman, which motivated her decision to pursue fertility preservation, should be respected. Nevertheless, special attention should be paid not to create false hope.
3. *For transgender individuals*: In case where providers lack adequate expertise, the patient should not be denied care but properly referred to another health professional. Parenting alternatives must be shared honestly, including all possible obstacles. Information should be tailored to each patient. This helps prevent both decision regret from premature refusal of fertility preservation and false hope of guaranteed offspring (Figure 2).

Fertility preservation techniques are advancing rapidly and will continue to raise ethical challenges and new dilemmas. Future research should explore how to address these issues and identify barriers in specific contexts. This can help integrate ethics more effectively into clinical fertility preservation practice.

## Data Availability

The original contributions presented in the study are included in the article/Supplementary Material, further inquiries can be directed to the corresponding author.
